# Type 2 diabetes is associated with suppression of autophagy and lipid accumulation in β‐cells

**DOI:** 10.1111/jcmm.14172

**Published:** 2019-02-01

**Authors:** Jeff Ji, Maria Petropavlovskaia, Armen Khatchadourian, Jason Patapas, Julia Makhlin, Lawrence Rosenberg, Dusica Maysinger

**Affiliations:** ^1^ Department of Pharmacology and Therapeutics McGill University Montreal QC Canada; ^2^ Department of Surgery McGill University Montreal QC Canada

**Keywords:** autophagy, islets, LAMP2, PLIN2, TFEB

## Abstract

Both type 2 diabetes (T2D) and obesity are characterized by excessive hyperlipidaemia and subsequent lipid droplet (LD) accumulation in adipose tissue. To investigate whether LDs also accumulate in β‐cells of T2D patients, we assessed the expression of PLIN2, a LD‐associated protein, in non‐diabetic (ND) and T2D pancreata. We observed an up‐regulation of PLIN2 mRNA and protein in β‐cells of T2D patients, along with significant changes in the expression of lipid metabolism, apoptosis and oxidative stress genes. The increased LD buildup in T2D β‐cells was accompanied by inhibition of nuclear translocation of TFEB, a master regulator of autophagy and by down‐regulation of lysosomal biomarker LAMP2. To investigate whether LD accumulation and autophagy were influenced by diabetic conditions, we used rat INS‐1 cells to model the effects of hyperglycaemia and hyperlipidaemia on autophagy and metabolic gene expression. Consistent with human tissue, both LD formation and PLIN2 expression were enhanced in INS‐1 cells under hyperglycaemia, whereas TFEB activation and autophagy gene expression were significantly reduced. Collectively, these results suggest that lipid clearance and overall homeostasis is markedly disrupted in β‐cells under hyperglycaemic conditions and interventions ameliorating lipid clearance could be beneficial in reducing functional impairments in islets caused by glucolipotoxicity.

## 1 INTRODUCTION

T2D is characterized by β‐cell failure, insulin resistance, hyperglycaemia, and hyperlipidaemia. β‐cells are nutrient sensors that regulate insulin secretion in response to elevated levels of glucose and lipids.^1^ Nutrient overload is viewed as the main cause of insulin resistance that increases the demand on β‐cells to secrete more insulin. If β‐cells fail to adapt to insulin resistance and become dysfunctional, glucose intolerance and overt diabetes develops.^2^ Evidence suggests that excessive lipids and the ensuing lipotoxicity play a major role in these processes by promoting insulin resistance and β‐cell dysfunction.^3^ However, the exact impact of lipid overload on β‐cell dysfunction remains poorly understood.

LDs are dynamic organelles implicated in metabolic disorders including obesity and T2D, which are marked by abnormal LD accumulation.^4^, ^5^ Structurally, LDs are natural micelles composed of a hydrophobic core containing triglycerides, cholesterol esters and a corona made of phospholipids that can serve as lipid storage sites and as a platform for the recruitment of signalling molecules.^6^, ^7^ Perilipin associated proteins (PAT) coat and stabilize the surface of LD by reducing lipases access to the lipid core. Perilipin‐2 (PLIN2) is the main housekeeping LD protein that is ubiquitously expressed and used as a marker for LDs in many human and animal tissues.^8^ PLIN5 is highly expressed in oxidative tissues and plays multiple metabolic roles such as fatty acid (FA) mobilization and has been shown to regulate post‐prandial insulin secretion in β‐cells.^9^, ^10^ High cellular lipid uptake (eg, post‐prandial period) increases PLIN2 (and other PAT proteins) expression and triggers their recruitment to the LD surface.^11^ These events temporarily reduce the cytosolic FA concentration. However, chronic lipid overload can lead to the formation of abnormally large and unstable LDs prone to FA spillover, which could lead to endoplasmic reticulum (ER) stress, induction of apoptosis and insulin resistance in β‐cells.^12^, ^13^ The timely removal of excessively ‘leaky’ LDs is therefore necessary to prevent lipotoxicity in β‐cells. Aside from cytosolic lipase activity, lipophagy is emerging as an important mechanism for LD degradation using the autophagic pathway^14^, ^15^ and is suggested as a general mechanism for degrading LD in diverse cell populations.^16^, ^17^, ^18^, ^19^

Targeted inactivation or repression of autophagy in β‐cells has been shown to cause diabetes in animal models. β‐cell–specific *Atg7* knockout led to islet degeneration in mice, accumulation of protein aggregates and decreased insulin production.^20^ Similarly, β‐cell–specific Tsc‐2 knockout, which caused mTORC1 hyperactivation and repression of autophagy, increased mitochondrial oxidation and ER stress, resulting in β‐cell failure.^21^ mTORC1 is a central kinase responsible for regulating many aspects of metabolism, energy utilization and cell growth in response to nutrient abundance within the cell. A direct effect of mTORC1 activity on LD formation in rat islet cells has been previously reported.^22^ mTORC1 inhibits autophagy partly through phosphorylation of transcription factor EB (TFEB) which prevents its nuclear translocation. During starvation, mTORC1 is suppressed and TFEB translocates to the nucleus and up‐regulates genes involved in autophagic and lysosomal production.^23^ TFEB is necessary for lipid degradation in the liver^24^ but its role in human pancreatic islets in the context of T2D has not been reported.

The goal of this study was to investigate the impact of T2D on LDs, autophagy and islet metabolism by assessing the expression and localization of PLIN2, TFEB, lysosome‐associated membrane protein‐2 (LAMP2) and genes associated with metabolism, oxidative stress, apoptosis and mitochondrial function in human pancreatic tissue from normal and T2D subjects. We have suggested that nutrient overload in diabetes causes LD accumulation due to decreased TFEB activation and suppression of autophagy and tested this hypothesis in vitro, using the rat insulinoma β‐cell line INS‐1.

## 2 MATERIALS AND METHODS

### 2.1 Human pancreatic tissue

Adult human pancreata were obtained from Quebec Transplant with prior consent for research use. Pancreatic tails were preserved in RNAlaterTM (Qiagen, Toronto, ON, Canada) for RNA extraction or fixed in 10% formalin (Fisher Scientific, Ottawa, ON, Canada) and paraffin‐embedded for immunolabelling (Pathology Unit, Montreal General Hospital, Montreal, Quebec, Canada). Donor information is summarized in Table S1. The study consisted of 22 ND and 17 type 2 diabetic patients.

### 2.2 Cell culture

INS‐1 rat insulinoma cells (AddexBio, San Diego, CA, USA) were cultured in RPMI‐1640 media containing 11.1 mmol/L [GLU], 2 mmol/L L‐glutamine, 10 mmol/L HEPES, 1 mmol/L sodium pyruvate, 2 g/L sodium bicarbonate, 10% FBS, 50 μmol/L 2‐mercaptoethanol, 1% penicillin‐streptomycin (Invitrogen, Waltham, MA, USA) and maintained at 37°C with 5% CO_2_.

### 2.3 Stable EGFP‐TFEB transfection of INS‐1 cells

INS‐1 cells were seeded in 6‐well plates (Starstedt, Montreal, QC, Canada) and transfected with pEGFP‐N1‐TFEB (CMV promoter, neomycin resistance) using Lipofectamine 2000 (FischerScientific) in culture medium for 48 hours. The medium was supplemented with 400 μg/mL geneticin (Sigma, Oakville, ON, Canada) to select for resistant cells and subsequently for single colonies by reseeding into 96‐well plates. EGFP‐positive clones showing functional TFEB translocation when starved in HBSS for 1 hour at 37°C were cultured with 200 μg/mL geneticin in the medium.

### 2.4 FA/BSA complex preparation

Oleic acid (OA) (Sigma) and palmitic acid (PA) (Sigma) were dissolved in Krebs‐Ringer bicarbonate buffer complexed with 5% fatty‐acid free BSA (Sigma) under gentle heating and stirring and sterile‐filtered through a 0.22 μm filter. FA concentration was quantified using Wako HR series NEFA‐HR(2) according to manufacturer instructions.

### 2.5 qRT‐PCR

For RNA extraction, human pancreatic samples stored at −80°C in RNAlater were homogenized in RLT buffer and processed in Qiacube^TM^ (Qiagen, Toronto, ON, Canada) using RNEasy mini kit according to manufacturer protocol. Quality and integrity of RNA was assessed by 1.5% agarose gel electrophoresis. For RNA extraction in cultured cells, INS‐1 were seeded at 2 000 000 cells in 150 mm plates (Sigma) and 48 hours after seeding were exposed to 5 mmol/L or 30 mmol/L [GLU] with or without 500 μmol/L OA, PA or 250 μmol/L OA + 250 μmol/L PA for 24 hours. Cells were lysed in RLT buffer and processed as above. Equal amounts of RNA, based on OD_260_, were reverse transcribed using oligo‐dT primers and Omniscript RT kit (Qiagen). One microlitre of cDNA was used for a 20 μL qPCR reaction performed with IQ^TM^ SYBR^®^ Green Supermix (Bio‐Rad, Mississauga, ON, Canada) in CFX96^TM^ Real‐Time System (Bio‐Rad) and primer pairs shown in Table S2. Multiple plates of experimental data, run with an inter‐plate calibrator, were combined into gene studies using glyceraldehyde 3‐phosphate dehydrogenase (GAPDH), β‐actin and succinate dehydrogenase complex flavoprotein (SDHA) as reference genes in human samples and β‐actin and β‐tubulin in INS‐1 samples (all M < 0.5). Fold change in gene expression (∆∆Ct) were obtained using the data analysis software CFX Manager3.1.

### 2.6 Immunohistochemistry

Tissue sections (5 μm) of pancreatic samples were deparaffinized in xylene and rehydrated in ethanol‐water mixtures. Antigen retrieval was performed in 95°C citrate buffer (10 mmol/L, 0.05% Tween‐20, pH 6.0) for 40 minutes and was followed by blocking in 2% horse serum/10% goat serum/1 mmol/L HEPES/0.1% sodium azide/0.3% Triton X‐100 in HBSS for 2 hours. Incubation with primary antibodies (Table S3) at 4°C overnight was followed by secondary antibodies, staining of nuclei in Hoechst 33342 or DAPI and mounting using Aqua‐Poly/Mount (Polysciences, Warrington, PA, USA). Quantification was performed in ImageJ by assessing the average fluorescence intensity normalized to the area and expressed as arbitrary units.

### 2.7 Lipid droplet staining and quantification

INS‐1 cells were seeded onto poly‐d‐lysine coated coverslips and incubated for 48 hours before treatment with 500 μmol/L OA in normoglycaemic (5 mmol/L [GLU]) or hyperglycaemic (30 mmol/L [GLU]) medium for 24 hours. Torin‐1 (Tocris, Bristol, UK) was added 3 hours before and with the 24 hours treatment. Cells were fixed in 2% PFA in PBS+ (with 0.5 mmol/L MgCl_2_, 1 mmol/L CaCl_2_) for 10 minutes at room temperature (RT). After washing with PBS+ cells were labelled with 20 μmol/L BODIPY 493/503 and 10 μmol/L Hoechst 33342 for 10 minutes at RT and washed with PBS+. Coverslips were mounted onto microscope slides with non‐hardening EverBrite Mounting medium (Biotium, Fremont, CA, USA) and the edges sealed with nail polish.

### 2.8 TFEB translocation test

INS‐1‐TFEB‐EGFP cells seeded in 24‐well plates (Starstedt) were treated in 5 mmol/L or 30 mmol/L [GLU] in the presence or absence of 500 μmol/L OA, PA or 250 μmol/L OA + 250 μmol/L PA for 48 hours. After treatment, cells were starved for 1 hour in HBSS, fixed in 2% PFA for 10 minutes at RT and washed twice with PBS+. After labelling nuclei with 10 μmol/L Hoechst 33342, cells were imaged by fluorescence microscopy and the images were analysed using ImageJ. Four fields were randomly chosen per well and EGFP‐positive cells were randomly chosen per field. TFEB‐EGFP nuclear translocation was assessed as the ratio of the area of normalized EGFP fluorescence intensity in the nucleus over the cytosol. A higher ratio indicates greater nuclear TFEB‐EGFP translocation.

### 2.9 Western blot

After treatment, total cellular protein was extracted using RIPA buffer containing protease inhibitors. Protein concentration was quantified using the Pierce BCA Protein Assay (ThermoFisher, Waltham, MA, USA). Proteins were subjected to SDS‐PAGE and transferred onto PVDF membrane. Membranes were blocked with 5% milk/TBST and incubated with primary antibodies followed by horseradish peroxidase‐conjugated secondary antibodies. Blots were developed using Clarity^TM^ Western ECL Substrate (BIO‐RAD, Hercules, CA, USA) and imaged using Amersham Imager 600 (GE Healthcare, Saint‐Laurent, QC, Canada).

### 2.10 Immunocytochemistry

After treatment, cells were fixed in 2% PFA in PBS+ (with 0.5 mmol/L MgCl_2_, 1 mmol/L CaCl_2_) for 10 minutes at RT and permeabilized with 0.1% Triton X‐100 in PBS+. Cells were blocked with 10% goat serum and incubated in primary antibody overnight at 4°C and incubated in secondary antibody for 1 hour at RT. Coverslips were mounted onto microscope slides using Aqua Poly/Mount.

### 2.11 Cell count and MTT

Cells were seeded into 24‐well plates (Starstedt) at 150 000 cells per well for 24 hours treatment or 75 000 cells per well for 48 hours treatment, 24 hours post‐seeding, cells were incubated in treatment media for 24 hours/48 hours. Cell count analysis: Cells were incubated with 10 μmol/L Hoechst 33342 for 10 minutes followed by imaging using a fluorescence microscope with a 4X objective. Four images per well were taken to represent the average number of cell in the well. The number of nuclei per field were counted using ImageJ. MTT assay 500 ug/mL MTT (Sigma) was added to cells and incubated for 40 minutes at 37°C. After incubation, the media were aspirated and DMSO was added to each well to dissolve formazan crystals. The absorbance was read at 595 nm using a plate reader.

### 2.12 Microscopy

Confocal imaging was performed with Zeiss LSM 510 NLO inverted confocal microscope using a Plan Achromat 63X/1.4 Oil DIC objective (Carl Zeiss Canada Ltd., Toronto, ON, Canada). All images were acquired at a resolution of 1024 × 1024 pixels (*x*,*y*). Fluorescence microscopy was performed with a Leica DMI4000 inverted microscope (Leica Microsystems Inc, Concord, ON, Canada) using a 4×, 10× or 63× oil objective captured using a Leica DFC345 FX camera (Leica Microsystems Inc).

### 2.13 Statistical analysis

All data are expressed as means ± standard error of the mean (SEM). Statistical differences were analysed by either student's *t* test or two‐way analysis of variance (ANOVA) followed by post‐hoc Tukey's test. Statistical significance was considered at *P* < 0.05. Statistical analysis was done using R v3.2.2.

## 3 RESULTS

### 3.1 PLIN2 abundance is markedly increased in human pancreata in T2D

**Figure 1:**
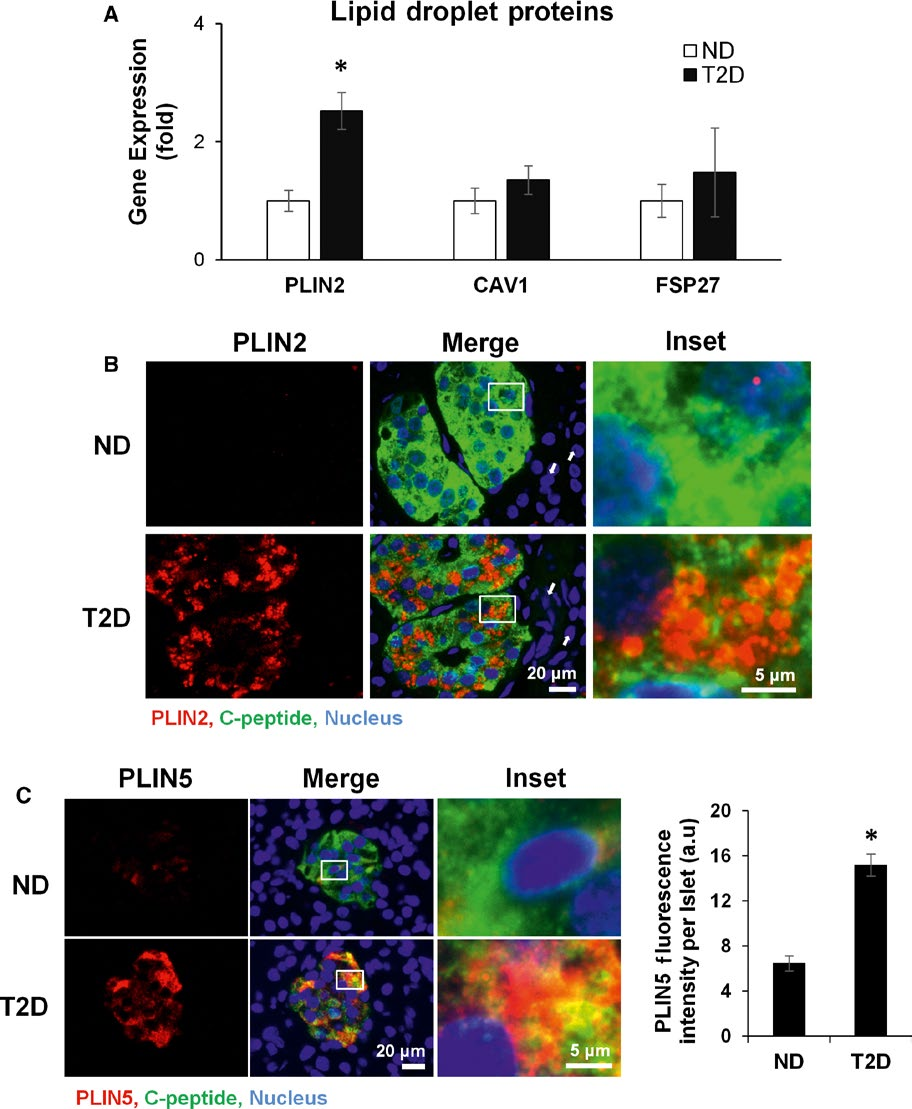
Expression of Lipid Droplet markers in the human pancreas. (A) Gene expression analysis of lipid droplet regulation genes from pancreatic tails (CAV1 = caveolin 1, PLIN2 = perilipin 2, FSP27 = fat specific protein 27). ND = 11 patients, T2D = 10 patients, graph represents average fold change ± standard error of the mean (SEM) in gene expression over ND. (B) Pictomicrograph of sections double immunolabelled with PLIN2 (red), C‐peptide (green) and stained with Hoechst 33342 (blue). Images are representative from four different donors per group. White arrows point to acinar tissue (exocrine tissue) which did not up‐regulate PLIN2. (C) Photomicrograph and fluorescence quantification of sections labelled with PLIN5 (red), C‐peptide (green) and stained with Hoechst 33342 (blue). Quantified from >21 islets from two different donors per group. Statistical analysis was evaluated by Student's *t* test and significance is indicated by * (compared to ND), *P < 0.05

We assessed whether T2D leads to changes in LD‐associated proteins: PLIN2, caveolin (CAV1) and fat specific protein 27 (FSP27) by qRT‐PCR (Figure 1A). PLIN2 mRNA was significantly up‐regulated in T2D (2.5 ± 0.3 ↑ fold increase, *P* < 0.001) (Figure 1A). However, there was no significant difference in gene expression of other LD‐associated proteins – CAV1, which plays an important role in cholesterol transport, lipogenesis and LD biogenesis,^25^ or FSP27, which promotes LD growth by lipid exchange and fusion.^26^

Immunostaining of pancreatic sections for the LD‐associated proteins showed a marked increase in PLIN2 protein in T2D islets, and demonstrated its predominant localization in β‐cells based on co‐staining with C‐peptide (Figure 1B), whereas it was undetectable in the acinar tissue. PLIN5 was also up‐regulated in β‐cells from T2D donors (Figure 1C). This abnormal accumulation of PLIN2 and PLIN5 and, by extension, of LDs in β‐cells is similar to that in hepatocytes in fatty liver disease or macrophages in atherosclerosis, where it is usually linked to the abnormal lipid catabolism.^27^ This prompted us to investigate whether the observed LD accumulation in T2D is accompanied and perhaps caused by an impaired processing of lipids in β‐cells.

### 3.2 TFEB activation is suppressed and LAMP2 abundance is reduced in T2D

**Figure 2:**
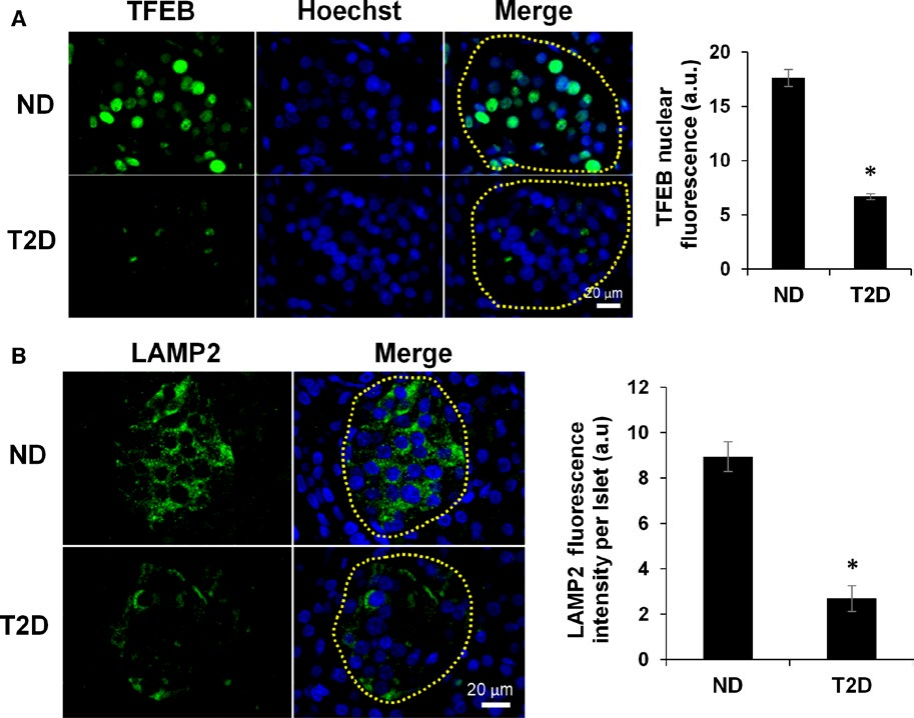
Autophagy markers in human islets. (A) Representative images of TFEB immunolabelling in ND, and T2D patients and quantification of TFEB nuclear fluorescence of cells within islets. Islets regions (encircled) were traced from bright‐field images based on morphology and nuclei were stained with Hoechst 33342. Quantifications are from ND = 8 patients (471 cells measured), T2D = 6 patients (416 cells measured). (B) Representative images of LAMP2 immunolabelling in ND and T2D patients and quantification of LAMP2 fluorescence within the islet. Quantifications are from ND = 6 patients (48 islets measured), T2D = 6 patients (61 islets measured). Statistical analysis was evaluated by Student's *t* test and significance is indicated by * (compared to ND), *P < 0.05, error bars = SEM

We evaluated the homeostatic clearance of lipids by autophagy by focusing on the activation of TFEB. TFEB nuclear translocation gauged by immunofluorescence was significantly decreased in islets from T2D donors compared to ND (3 ± 0.01 ↓, *P* < 0.001) (Figure 2A), which suggested down‐regulation of autophagic genes. Since TFEB controls lysosome‐related genes, we quantified LAMP2 protein, a structural marker for lysosomes and found that it was significantly decreased in T2D compared to ND (3.3 ± 0.2 ↓, *P* < 0.001, Figure 2B). These data support our hypothesis that T2D‐associated reduction in islet TFEB and subsequently in lysosomal activity led to suppression of autophagy.

### 3.3 Expression of islet metabolism, redox and apoptosis related genes is altered in T2D

**Figure 3:**
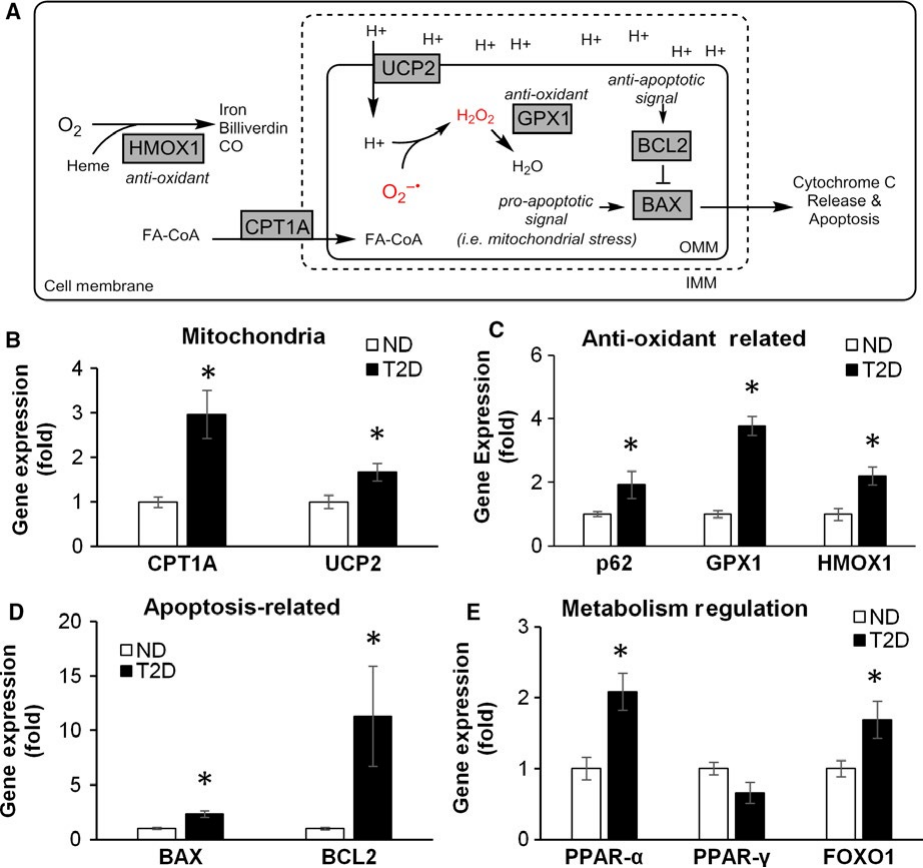
Metabolism, redox and apoptosis related gene expression in human pancreata. (A) Schematic of the genes assayed below. CPT1A = carnitine palmitoyltransferase 1A couples carnitine to acyl groups which enables fatty acids to enter the mitochondria and undergo β‐oxidation. UCP2 = uncoupling protein 2 enables protons to flow back into the mitochondria matrix without oxidative phosphorylation which generates heat instead of ATP, p62 = sequestome 1, delivers cargo to autophagosomes for degradation, regulates Nrf2 activity by binding with Keap1. HMOX1 = heme oxygenase 1, converts denatured heme, oxygen and NADPH to Billiverdin, carbon monoxide and iron. It is an anti‐oxidant gene up‐regulated by oxidative stress. GPX1 = glutathione peroxidase 1 is an anti‐oxidant enzyme which scavenges hydrogen peroxide. BAX = BCL2 associated X, BCL2 = BCL2 are respectively pro‐apoptotic and anti‐apoptotic regulators. FOXO1 = forkhead box protein 1, PPAR‐α = peroxisome proliferator activated receptor‐α, PPAR‐γ = peroxisome proliferator activated receptor‐γ are transcription factors which regulates many facets of metabolism. Gene expression analysis of (B) mitochondria, (C) oxidative stress/oxidant defense, (D) apoptosis‐related, (E) metabolism regulation related genes from pancreatic tails. Graphs represent average fold increase ± standard error of the mean (SEM) in gene expression between groups over ND. N = 11 patients for ND, N = 10 patients for T2D. Statistical analysis was evaluated by Student's *t* test and significance is indicated by * (compared to ND), *P < 0.05

Since T2D has a major impact on islet metabolism, we examined the expression of pancreatic genes regulating mitochondrial function, apoptosis and oxidative stress, as illustrated in Figure 3. Among mitochondrial genes, there was a significant up‐regulation in the expression of Carnitine palmitoyltransferase 1A (CPT1A), a crucial FA transporter on the surface of mitochondria (3 ± 0.5 ↑, *P* < 0.01, Figure 3B). This suggests an increase in β‐oxidation to combat the increased lipid load. Elevated β‐oxidation would increase the transmembrane potential of the mitochondria. Accordingly, Uncoupling protein 2 (UCP2) was also up‐regulated in T2D (1.7 ± 0.2 ↑, *P* < 0.05) suggesting an increase in non‐ATP coupled proton leak (Figure 3B). UCP2 up‐regulation was previously reported in response to oxidative stress in β‐cells.^28^ We next evaluated expression of several genes related to anti‐oxidant defence and apoptosis and found significant up‐regulation of glutathione peroxidase 1 (GPX1, 3.8 ± 0.3 ↑, *P* < 0.001) and heme oxygenase 1 (HMOX1, 2.2 ± 0.3 ↑, *P* < 0.01) in T2D pancreata (Figure 3C), which correlated with an increased immunofluorescence for Nrf2 (Figure S1), a transcription factor regulating GPX1, HMOX1 and other genes involved in oxidant defense and redox signalling.^29^ One of these genes, p62 (SQSTM1), is directly involved in autophagy and is used as a measure of autophagy flux,^30^ was also up‐regulated in T2D (Figure 3). Expression of anti‐apoptotic gene BCL2 was strongly increased (11.3 ± 4.6 ↑, *P* < 0.05) (Figure 3D), however pro‐apoptotic gene BAX was also up‐regulated (2.3 ± 0.3 ↑, *P* < 0.001). Finally, we examined transcription factors linked to metabolism. T2D leads to significant changes in the expression of transcription factors forkhead box protein 1 (FOXO1, 1.7 ± 0.3 ↑, *P* < 0.05), and peroxisome proliferator‐activated receptor‐alpha (PPAR‐α, 2.1 ± 0.3 ↑, *P* < 0.01) (Figure 3E), while PPAR‐γ expression did not differ between T2D and ND. Together, these changes in mRNA level in mitochondria, anti‐ROS defense and apoptosis‐associated genes in the pancreata of T2D subjects, suggest the activation of compensatory mechanisms to withstand metabolic stress and that the effects of diabetes were likely still within the adaptable range and have not reached the stage of irrevocable damage. To assess the morphology and architecture of the islets, we immunostained for glucagon (α‐cells), insulin (β‐cells) and somatostatin (δ‐cells) using sections from several patients in both ND and T2D groups. Both ND and T2D islets contained similar distribution of α‐, β‐ and δ‐cells (Figure S2), suggesting that the altered metabolic profiles in T2D subjects were not caused by gross morphological changes.

### 3.4 Hyperglycaemia increases LD and Plin2 in INS‐1

**Figure 4:**
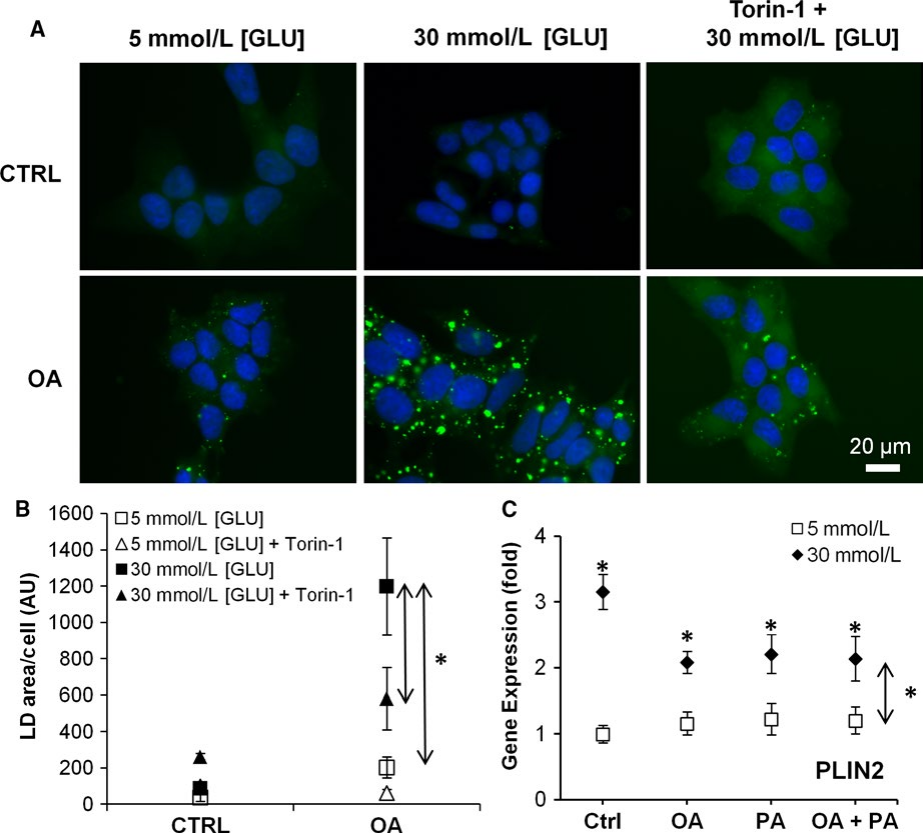
Effect of hyperglycaemia and hyperlipidaemia on LD and LD‐associated proteins in INS‐1. (A) Representative image and (B) quantification of INS‐1 pre‐treated with 1 μmol/L Torin‐1 for 3 h before treatment with 5 mM (normoglycaemic) [GLU] or 30 mM [GLU] (hyperglycaemic) medium ±500 μM oleic acid (OA) for 24 h and stained for LD with BODIPY 493/503 (green) and Hoechst 33342 (blue). LD was quantified as the total area of LD per cell and represents the mean from three independent experiments. (C) Gene expression analysis of INS‐1 Plin2 under 5 mmol/L [GLU], 30 mmol/L [GLU]), treated with 500 μmol/LM OA, 500 μmol/L palmitic acid (PA) or 250 μmol/L OA + 250 μmol/L PA. Graphs represent average fold increase ± standard error of the mean (SEM) in gene expression between groups from three independent experiments. Statistical analysis was evaluated by two‐way ANOVA followed by Tukey's test. Dual arrows * indicate a significant main effect due to hyperglycaemia (*P* < 0.05), * beside each point indicate significant difference (*P* < 0.05) compared to 5 mmol/L [GLU] Ctrl

Although pancreatic dysfunction in T2D and obesity is multifactorial and involves a host of metabolic and hormonal changes, two major contributors are hyperglycaemia and hyperlipidaemia. To better understand the observed changes in LDs and in gene expression in T2D pancreata, we examined the direct effects of hyperglycaemia and hyperlipidaemia on LD and autophagy in vitro using INS‐1 cells. 5 mmol/L glucose was taken as normoglycaemia and 30 mmol/L glucose as hyperglycaemia. Hyperlipidaemia was induced by 500 μmol/L OA, 500 μmol/L PA or 250 μmol/L oleic acid +250 μmol/L palmitic acid (OA+PA), as used by others.^22^, ^31^ This was the maximal dose which did not significantly reduce cell number or induce apoptosis (Figure S3A,B,E) yet it did increase mitochondrial metabolic activity relative to 5 mmol/L [GLU] control (Figure S3C,D). INS‐1 cells treated with OA under hyperglycaemia showed a considerable increase in the number of LD (fivefold increase in LD area compared to normoglycaemia, *P* < 0.01, Figure 4A,B). Furthermore, two‐way ANOVA analysis revealed significant synergistic effect (*P* < 0.05) between hyperglycaemia and OA treatment on LD area per cell. The increased LD storage due to hyperglycaemia was partially abolished by pre‐treatment with the mTOR inhibitor/autophagy inducer Torin‐1, which suggests that activation of autophagy could reduce lipid accumulation. In addition, we show that hyperglycaemia alone was the main factor leading to significant up‐regulation of Plin2 (*P* < 0.001, Figure 4C), while FA alone (normoglycaemic conditions) only slightly elevated Plin2 mRNA. Interestingly, FA treatment under hyperglycaemia lowered the effect of hyperglycaemia on Plin2 expression. This discrepancy between Plin2 mRNA and the LD number under FA+30 mM [GLU] may indicate that Plin2 is also regulated at the translational level and that Plin2 alone is not sufficient for LD formation and growth.

### 3.5 Hyperglycaemia inhibits TFEB nuclear translocation and down‐regulates autophagy

**Figure 5:**
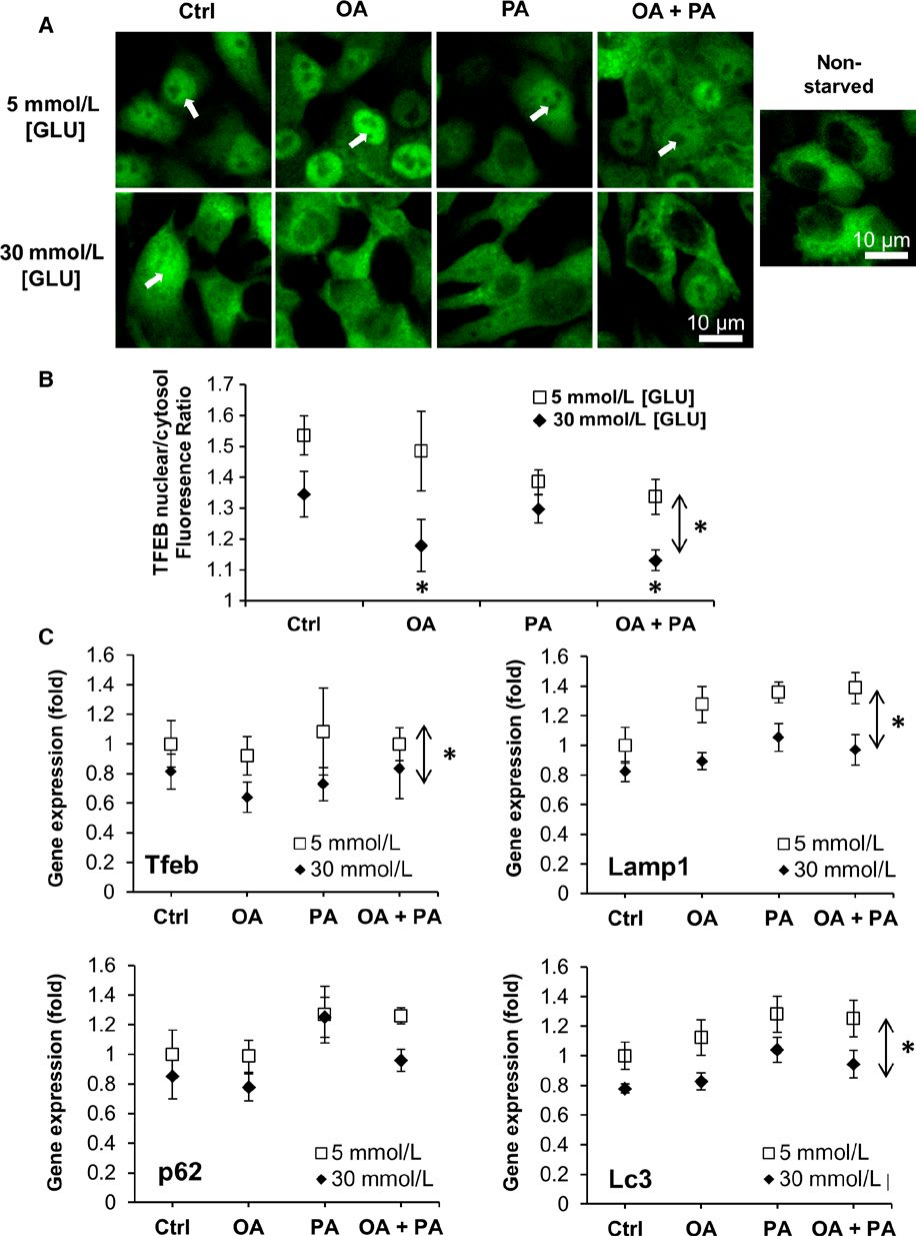
TFEB translocation and autophagy‐associated gene expression in INS‐1. (A) INS‐1‐TFEB‐EGFP was cultured for 48 h in normoglycaemia (5 mmol/L [GLU]), hyperglycaemia (30 mmol/L [GLU]) and treated with 500 μmol/L OA, 500 μmol/L PA, or 250 μmol/L OA + 250 μmol/L PA and starved for 1 h in HBSS to induce TFEB nuclear translocation. (A) Representative images, (B) quantification of nuclear/cytosolic TFEB fluorescence ratio. n > 50 cells from three independent experiments. White arrows point to TFEB nuclear translocation. (C) Gene expression analysis of INS‐1 Tfeb, Lamp1, Sqstm1 and Lc3 under 5 mmol/L [GLU]/30 mmol/L [GLU]), treated with 500 μmol/L OA, 500 μmol/L palmitic acid (PA) or 250 μmol/L OA +250 μmol/L PA. Graphs represent average fold increase ± standard error of the mean (SEM) in gene expression between groups from three independent experiments. Statistical analysis was evaluated by two‐way ANOVA followed by Tukey's test. Dual arrows * indicate significant main effect due to hyperglycaemia (*P* < 0.05), * beside each point indicate significant difference (*P* < 0.05) compared to 5 mmol/L [GLU] Ctrl

Given that TFEB nuclear translocation and LAMP2 expression are suppressed in the islets of T2D, we hypothesized that hyperglycaemia and hyperlipidaemia may inhibit TFEB nuclear translocation and autophagy. To test our hypothesis, INS‐1 cells stably transfected with TFEB‐EGFP were exposed to 5 or 30 mmol/L [GLU] in the presence and absence of FAs for 48 hours and then subjected to starvation to measure TFEB nuclear translocation. Treatment with OA and OA+PA under hyperglycaemic conditions significantly reduced TFEB nuclear translocation (Figure 5A,B). These results show that the combination of high glucose concentration with lipid overload inhibits TFEB activation. Hyperglycaemia lowered the expression of Tfeb, Lamp1, and Lc3 (Figure 5C), meaning that autophagy was down‐regulated at the transcriptional (TFEB), lysosomal (Lamp1) and autophagosome assembly (LC3) levels. Expression of p62, a scaffold protein known to bind ubiquitin and LC3 and degrade by autophagy,^30^ followed the same pattern as LC3 except for PA+30 mmol/L [GLU] when no down‐regulation by high glucose was observed. Since p62 protein accumulates when autophagy is inhibited, we used p62 accumulation (seen as aggregated puncta) as a measure of autophagic blockage. Chloroquine (inhibitor of autophagy) was used as a positive control for autophagy blockage (Figure S4A). PA+30 mmol/L [GLU] significantly increased p62 levels and aggregation (Figure S4A,B), while OA+PA+30 mmol/L [GLU] had a lesser effect. Inhibition of autophagy was further confirmed by a significant decrease in LC3BII/I ratio under hyperglycaemia (Figure S4C). Given that down‐regulation of autophagy is known to dysregulate insulin production and islet metabolism, we performed qRT‐PCR for Ins1 and Ins2, Ppar‐α, Ppar‐γ, Ucp2 in INS‐1 cells exposed to 5 or 30 mmol/L [GLU] with or without OA, PA, OA+PA (Figure S5). Under normoglycaemia, FA treatments stimulated a significant increase in insulin expression, whereas under hyperglycaemia FA had a smaller effect, suggesting that prolonged hyperglycaemia can reduce insulin production. This corroborates with the recently reported significant reduction in insulin expression in INS‐1 cells after 14‐day exposure to 22.4 mmol/L [GLU] due to methylation silencing of the insulin promoter.^32^

## 4 DISCUSSION

**Figure 6:**
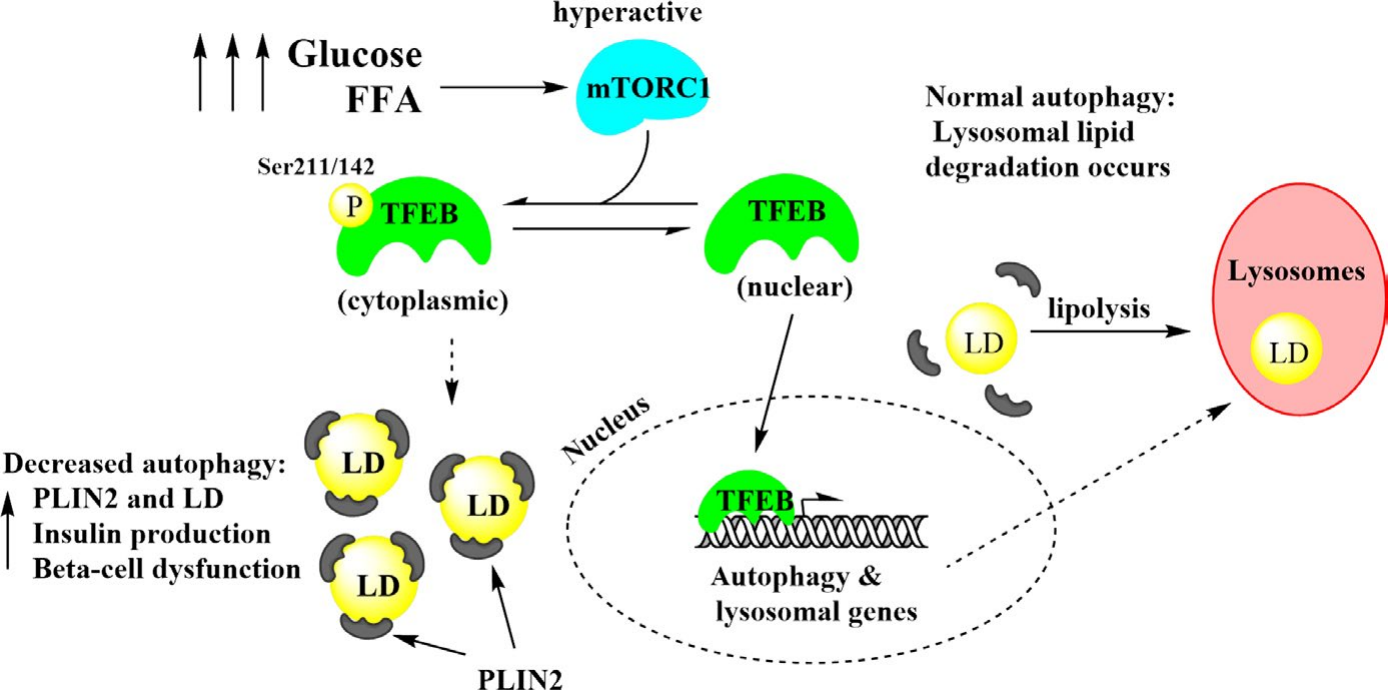
Schematic for dysregulated TFEB and lipid droplet accumulation during hyperglycaemia and hyperlipidaemia. Elevated glucose and FFA increase mTORC1 activity which leads to greater suppression of TFEB through inhibitory phosphorylation. As a consequence, LDs accumulate inside of β‐cells due to increased lipid load as well as decreased lipophagy due to the down‐regulation of autophagy and lysosomal biogenesis

Results from this study show that hyperglycaemia and hyperlipidaemia in humans and rat insulin‐producing cells (INS‐1) lead to: (1) an up‐regulation of LD‐associated protein PLIN2, (2) a significant decrease in TFEB activity and in lysosomal biomarker LAMP2, consistent with inhibition of autophagy and (3) dysregulation of genes implicated in lipid metabolism, mitochondrial function and cell survival. A schematic of the proposed mechanisms is illustrated in Figure 6.

Under physiological conditions, β‐cells preferentially use FA over glucose through β‐oxidation.^33^ We showed that hyperglycaemia up‐regulated PLIN2 and substantially increased OA‐induced LD formation. The primary function of LDs is to store lipids for energy but also to prevent acute lipotoxicity by sequestering otherwise toxic FA.^4^ In isolated rat β‐cells, the accumulation of LD was inversely proportional to the cytotoxicity for a given FA, for example palmitate lead to lower triglyceride accumulation and greater cell toxicity compared to oleate.^34^ However, nutrient‐induced LD accumulation is also associated with β‐cell dysfunction.^22^ Reduction in LD by PLIN2 knockdown in β‐cells lead to decreased ER stress, enhanced autophagy and decreased β‐cell apoptosis in diabetic animal models.^35^This is consistent with our observations of the changes in autophagy‐related proteins TFEB and LAMP2 and in the gene expression in T2D pancreata suggesting the activation of compensatory mechanisms to withstand metabolic stress. For instance, to reduce the ATP/ADP ratio under hyperglycaemic conditions, mitochondria can increase proton leakage through uncoupling proteins. We report an increase in both UCP2 and CPT1A expression in the pancreas of T2D patients which suggests an increase in both FA mitochondrial import and in β‐oxidation. We also observed a significant increase in mitochondrial metabolic activity in INS‐1 exposed to hyperglycaemic conditions after 24 and 48 hours.

In this study, PLIN2 protein was used as a LD marker to gauge the size of the LD pool. PLIN2 is actively degraded in the cytosol when it is not on the surface of LD.^36^ Interestingly, a number of studies report that PLIN2 has additional functions not directly related to lipid accumulation, such as mediating Wnt/LiCl signalling,^37^ activation of ER and unfolded protein response in β‐cells (UPR),^35^ and inhibition of glucose uptake through interactions with SNAP23.^38^ Expression of PLIN2 is up‐regulated by lipids,^39^ ROS,^40^ ER stress and UPR.^35^ We found that 30 mmol/L [GLU] alone was the strongest inducer of PLIN2 expression in INS‐1, whereas FA alone did not have an effect (Figure 4). We were surprised to see no effect of FA on PLIN2 mRNA in low glucose, contrary to the notion that PLIN2 expression is up‐regulated by lipids.^39^ However, the differing culturing glucose concentration and different treatment times complicates the comparison between our study and that of Faleck et al. 39

Up‐regulation of PLIN2 in T2D patients is likely caused by a combination of hyperglycaemia, hyperlipidaemia, inflammation and oxidative stress, among others. As a chronic phenomenon, prolonged accumulation of PLIN2 and LD can contribute to ER stress and β‐cell loss. Consistent with this, we show an increase in pro‐apoptotic gene BAX in T2D as well as up‐regulation of anti‐apoptotic gene BCL2 and oxidant defense genes (GPX1, HMOX1, NRF2) to counteract ROS induced by ER stress. The PLIN family of proteins are also known to regulate the activity of a multitude of LD associated proteins,^41^ including lipases such as ATGL^42^ and HSL.^43^ Future studies should examine the expression and activity of LD associated proteins such as lipid synthesis enzymes, lipases and trafficking proteins to completely characterize the regulation of lipid storage and breakdown in T2D.

Our data, from T2D samples and INS‐1 cells, are consistent with inhibition of autophagy by hyperglycaemia and hyperlipidaemia. Autophagy (and lipophagy) plays multiple roles in maintaining cellular homeostasis.^44^ TFEB is a master regulator of autophagic genes and lysosome biogenesis. A previous study has reported that mice fed a high‐fat diet have reduced TFEB mRNA levels in pancreatic islets.^45^ Our observation of reduced TFEB nuclear translocation and LAMP2 protein levels in T2D islets is consistent with previous findings of reduced LAMP2 and cathepsin‐B/D gene expression in islets from T2D patients.^46^ Reduction in TFEB activity as a consequence of diabetes is not limited to β‐cells. A recent study in cardiomyocytes has found reduced TFEB expression in both a mouse model of type 1 diabetes (Akita mice) and in obese patients.^47^

Active mTORC1 up‐regulates anabolic processes and suppresses autophagy. Vernier et al have previously demonstrated that hyperglycaemia and hyperlipidaemia lead to increased mTORC1 activity.^22^ Our data on inhibition of LDs in 30 mmol/L [GLU]+OA treated cells by Torin‐1 are consistent with those findings. As an extension, we found a decreased rate of starvation‐induced TFEB nuclear translocation in β‐cells pre‐treated with elevated glucose and FA. Hyperactivation of mTORC1 may be a general metabolic dysregulation due to nutrient overload. Rapamycin inhibition of mTORC1 reversed insulin resistance in adipose tissue, skeletal muscle and liver in hyperinsulinaemic rats.^48^ Importantly, rapamycin inhibition of mTORC1 greatly diminished LD accumulation in rodent β‐cells.^22^ Although abnormally active mTORC1 leads to deleterious diabetic‐like symptoms, normal mTORC1 function is still critical for β‐cell proliferation and metabolic function. Long‐term rapamycin treatment leads to worsened glucose intolerance, lipid accumulation in the liver and β‐cell loss in diabetic animals.^49^, ^50^ Whether increased TFEB activation can rescue β‐cell loss and improve insulin sensitivity has yet to be explored. In both genetic and diet‐induced models of obesity, TFEB overexpressing mice had lower blood glucose levels, improved glucose tolerance and decreased fat gain compared to WT, whereas TFEB‐knockout mice experienced increased fat gain.^24^ Interestingly, TFEB activity is increased during physical exercise and is necessary for the beneficial effects of exercise such as increased FA oxidation, insulin response and improved mitochondrial function in skeletal muscle.^51^

Nutrient overload is a new phenomenon at the evolutionary timescale and in this context Neel proposed a thrifty gene hypothesis^52^ to explain the adaptation of β‐cells in oscillating periods of low and high energy load. Periods of fasting would enable β‐cells to up‐regulate autophagy through TFEB nuclear translocation and recycle worn‐out organelles and accumulating lipids. A recent study on islet regeneration has shown fasting in diabetic mice lowers mTOR activity, promotes β‐cell neogenesis and reverses diabetic symptoms.^53^ Intermittent fasting proved beneficial in other diseases associated with impaired autophagy including Alzheimer's disease, excitotoxicity and in aging.^53^, ^54^, ^55^

Combined therapeutic approaches targeting TFEB and mTORC1 with intermittent fasting could be a rewarding direction in the intervention of T2D and other metabolic disorders. A limitation of our current study is the scarcity of human pancreatic tissue and the difficulty of obtaining freshly isolated islets. Instead, we used the well‐studied rat β‐cell line (INS‐1) to model the effects of hyperglycaemia on LD and autophagy in vitro. We used supraphysiological levels of glucose (30 mmol/L) to model hyperglycaemia in INS‐1 cells for two reasons: (1) rat diabetic models have two‐ to threefold higher circulating glucose compared to humans^56^, ^57^; (2) previous in vitro studies using INS‐1 cells employed ≥25 mmol/L glucose to model hyperglycaemia.^58^, ^59^ Having established an important connection between autophagy dysregulation and LD accumulation in our current study, future experiments should explore whether TFEB overexpression in β‐cells can rescue islet dysfunction in vivo. Complementary experiments with human islets could provide new directions for the therapeutic interventions utilizing lipophagy to normalize lipid homeostasis.

## CONFLICT OF INTEREST

The authors declare that they have no conflicts of interests.
