# Supplementary material for: Type 2 diabetes is associated with suppression of autophagy and lipid accumulation in β‐cells
Source: J Cell Mol Med. 2019 Feb 1;23(4):2890–900. doi: 10.1111/jcmm.14172 (PMC6433726; doi:10.1111/jcmm.14172)
Supplement: Supplementary file 1 [file JCMM-23-2890-s001.docx]

**Title: Type 2 diabetes is associated with suppression of autophagy and lipid accumulation in β-cells**

Jeff Ji^ƚ1^, Maria Petropavlovskaia^ƚ2^, Armen Khatchadourian^1^, Jason Patapas^2^, Julia Makhlin^2^, Lawrence Rosenberg^2^, Dusica Maysinger*^1^

*Jeff Ji and Maria Petropavlovskaia contributed equally to this work

^1^Department of Pharmacology and Therapeutics, McGill University, Montreal, QC, H3G 1Y6, Canada

^2^Department of Surgery, McGill University, Lady Davis Institute, Montreal, QC, H3T 1, Canada

* Corresponding author: Dusica Maysinger

Email: [dusica.maysinger@mcgill.ca](mailto:dusica.maysinger@mcgill.ca)

Phone: [(514) 398-1264](tel:%28514%29%20398-1264)

Fax: [(514) 398-6690](tel:%28514%29%20398-6690)

Department of Pharmacology and Therapeutics, McGill University, 3655 Promenade Sir William Osler, Montreal, QC, H3G 1Y6, Canada

**Contains:**

1. Supplementary Figure S1-S5

3. Supplementary Table 1-3

**Supplementary Figure S1**

Nrf2 is upregulated in pancreatic islets from T2D patients. (A) Photomicrograph of sections double immunolabeled with Nrf2, Insulin, and stained for the nucleus with Hoechst 33342. B) Quantification of Nrf2 fluorescence from ND=10 patients (48 islets measured), T2D=6 patients (43 islets measured). Statistical analysis was evaluated by student’s t-test and significance is indicated by * (compared to ND), * p<0.05, error bars = SEM.

**
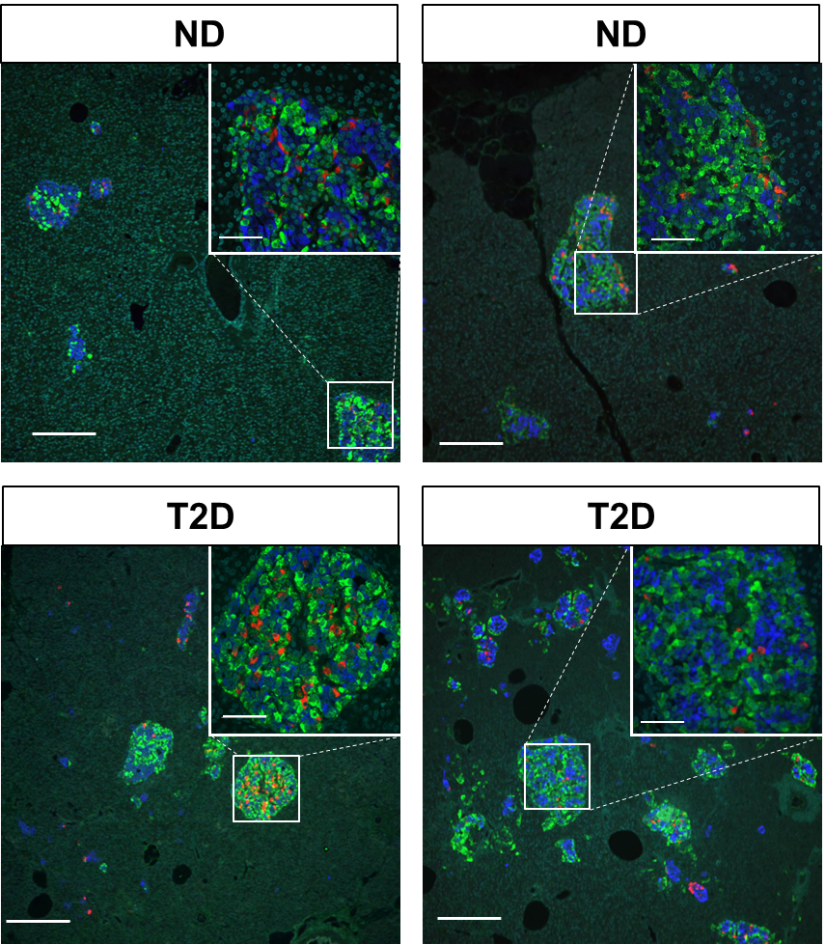
**

**Supplementary Figure S2**

Representative images of islet cells from two different ND, T2D pancreata. Insulin is shown in green, glucagon is shown in red, and nuclei are shown in blue. Overall, islets from both ND and T2D contained similar percentage of insulin and glucagon producing cells were similar. Scale bar = 200 μm, inset scale bar = 50 μm.

**Supplementary Figure S3**

Measurement of cell viability and apoptosis in INS-1. (A-B) Number of cells in culture after 24h (A) and 48h (B) treatment with 100 – 1000 µM oleic acid, palmitic acid or a combination in 5 mM [GLU] or 30 mM [GLU] medium. (C-D) Measurement of mitochondrial metabolic activity in similar conditions to A-B. Graphs represent average ± SEM from 2-3 independent experiments. Statistical analysis was evaluated by student’s t-test and significance is indicated by * p<0.05 compared to 5 mM [GLU] Control. (E) Western blot for procaspase-3 (inactive) and caspase-3 (active) in INS-1 cells. No active caspase-3 was detected. β-actin was used as a loading control.

**Supplementary Figure S4**

p62 accumulation and LC3B-I/II levels in INS-1 cells. (A) Representative images, (B) quantification of p62 fluorescence in INS-1 cultured for 48h in normoglycemia (5 mM [GLU]), hyperglycemia (30 mM [GLU]), and treated with 500 μM OA, 500 μM PA, 250 μM OA + 250 μM PA or 50 μM chloroquine overnight. White arrows indicate p62 accumulation. Graph represents average fold increase over control ± SEM between groups from 2 independent experiments. Statistical analysis was evaluated by student’s t-test and significance is indicated by * p<0.05 compared to 5 mM [GLU] Control. (C) Western blot of INS-1 LC3B-I/II. Increased LC3B-II/LC3B-I indicates greater autophagic activity. Graph represents average LC3B-II/LC3B-I ratio from 2 independent experiments. β-actin was used as a loading control.

**
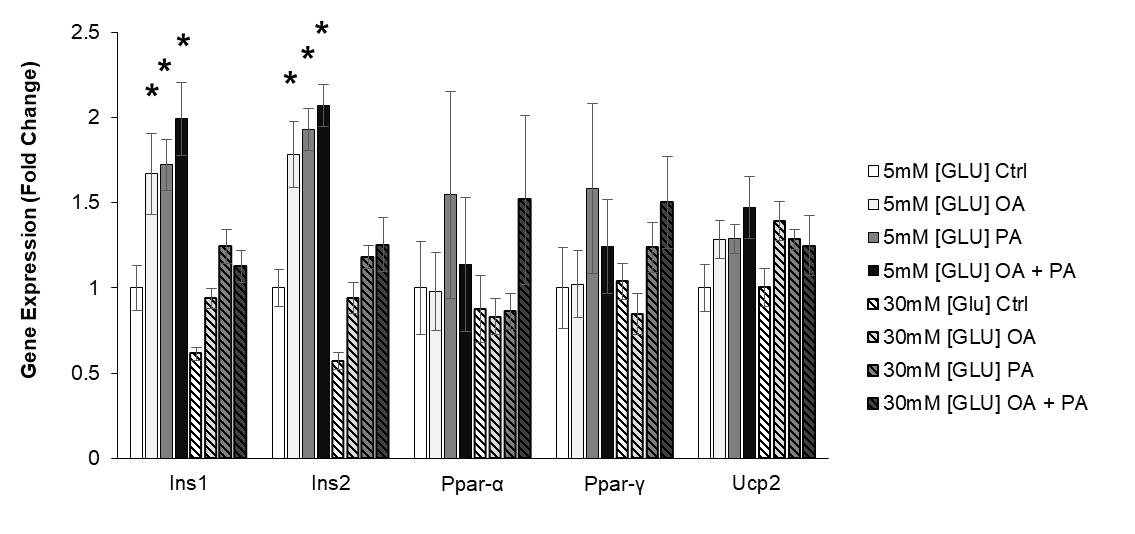
**

**Supplementary Figure S5**

Gene expression analysis in INS-1 of metabolism-associated genes under normoglycemic (5 mM [GLU]), hyperglycemic (30 mM [GLU]), treated with 500 μM oleic acid (OA), 500 μM palmitic acid (PA), or 250μM oleic acid (OA) + 250 μM palmitic acid (PA). Graphs represent average fold increase ± standard error of the mean (SEM) in gene expression between groups from 3 independent experiments. Statistical analysis was evaluated by two-way ANOVA followed by TukeyHSD. * indicate significant difference (p<0.05) compared to 5 mM [GLU] Ctrl.

**Supplementary Table 1. Patient information**

| **Sample** | **Age** | **Sex** | **Weight (kg)** | **Height (cm)** | **BMI** | **Medical History** | **Medication** | **Cause of death** |
| --- | --- | --- | --- | --- | --- | --- | --- | --- |
| NDN1 | 74 | - | 61 | 165 | 22.4 | na | na | Cranial trauma |
| NDN2 | 50 | M | 70 | 178 | 22.1 | na | na | Stroke |
| NDN3 | 35 | F | 48 | 148 | 21.8 | na | na | Stroke |
| NDN4 | 26 | F | 70 | 170 | 24.2 | na | na | Anoxia |
| NDN5 | 52 | F | 53 | 162 | 20.2 | na | na | Cerebral hemorrhage |
| NDN6 | 72 | F | 59 | 163 | 22.3 | na |  | Trauma |
| NDN7 | 14 | F | 50 | 162 | 19.1 | na | na | Trauma |
| NDN8 | 56 | F | 52 | 160 | 20.3 | na | na | Cerebrovascular accident |
| NDN9 | 40 | M | 59 | 180 | 18.2 | na | na | Trauma |
| NDN10 | 63 | F | 56 | 158 | 22.4 | na | na | - |
| NDO1 | 62 | F | 82 | 160 | 31.8 | na | na | Cerebral hemorrhage |
| NDO2 | 51 | M | 118 | 180 | 36.4 | na | na | Cerebral hemorrhage |
| NDO3 | 70 | F | 90 | 160 | 35.2 | na | na | Cerebral hemorrhage |
| NDO4 | 60 | F | 90 | 157 | 36.5 | na | na | Stroke |
| NDO5 | 48 | F | 74 | 157 | 30 | na | na | Anoxia |
| NDO6 | 57 | F | 95 | 160 | 37.1 | na | na | Cerebral hemorrhage |
| NDO7 | 12 | - | 52 | 168 | 18.4 | na | na | Cerebral hemorrhage |
| NDO8 | 55 | M | 75 | 165 | 27.5 | na | na | Cerebral edema |
| NDO9 | 35 | F | 122 | 165 | 44.8 | na | na | Subarachnoid hemorrhage |
| NDO10 | 68 | M | 95 | 168 | 33.7 | na | na | Sub-dural hemorrhage |
| NDO11 | 64 | F | 90.5 | 150 | 40.2 | na | na | Cerebral hemorrhage |
| NDO12 | 71 | M | 94 | 188 | 26.6 | na | na | Trauma |
| T2DN1 | 54 | F | 59 | 158 | 23.6 | T2D, hypertension, COPD, hyperlipidemia, smoker | Glyburide, metformin | Cerebral hemorrhage |
| T2DN2 | 54 | M | 80 | 178 | 25.2 | T2D, hypertension, hyperlipidemic | Anti-hyperglycemic, diet | Trauma |
| T2DN3 | 63 | M | 70 | 170 | 24.2 | T2D, smoker, alcoholic | Diet | Trauma |
| T2DN4 | 60 | M | 73 | 170 | 25.3 | T2D, hypertension, hyperlipidemic, smoker, alcoholic | Diet | Anoxic enchephalopathy |
| T2DN5 | 64 | M | 64 | 170 | 22.1 | T2D | Metformin | Cerebral hemorrhage |
| T2DN6 | 63 | M | 70 | 170 | 24.2 | T2D, smoker, alcoholic | - | Trauma |
| T2DN7 | 64 | M | 64 | 170 | 22.1 | T2D | Metformin | Cerebral hemorrhage |
| T2DN8 | 54 | F | 59 | 158 | 23.6 | 2D, hypertension, COPD, hyperlipidemia, smoker | Glyburide, metformin | Cerebral hemorrhage |
| T2DO1 | 54 | F | 83 | 165 | 30.5 | T2D | Insulin | Abdominal trauma |
| T2DO2 | 69 | F | 138 | 177 | 44.0 | T2D | - | Cerebral hemorrhage |
| T2DO3 | 60 | M | 113 | 173 | 37.9 | T2D, hypertension, smoker | Metformin | Cerebral anoxia |
| T2DO4 | 66 | F | 81 | 154 | 34.2 | T2D, manic depression, smoker | Metformin, glyburide | Sub-arachnoid hemorrhage |
| T2DO5 | 66 | M | 95 | 170 | 32.8 | T2D | Metformin | Anoxia |
| T2DO6 | 54 | F | 77 | 160 | 30.1 | T2D, hypertension | - | Cerebrovascular accident |
| T2DO7 | 75 | M | 76 | 166 | 27.58 | T2DM, Smoker, | Glyburide | Cerebral hemorrhage |
| T2DO8 | 53 | M | 106.4 | 156 | 43.7 | T2D | Norepinephrine bitartrate | AVC hemorrhage |
| T2DO9 | 65 | F | 86.7 | 157 | 35.2 | T2D | Metformin | Anoxia |

**Supplementary Table 2. Primers used in this study**

| **Primers for Human Genes** | | | | |
| --- | --- | --- | --- | --- |
| **Gene** | **Reference** | **Forward** | **Reverse** | **Amplicon(bp)** |
| PLIN2 | NM_001122 | GCTGAGCACATTGAGTCACG | GCATTGCGGAACACTGAGTAGA | 172 |
| ACTB | NM_001101 | TTCCTGGGCATGGAGTCCTGT | CTTGATCTTCATTGTGCTGGGTGC | 188 |
| BAX | NM_138761 | TGCTTCAGGGTTTCATCCAGGA | CTGTCCAGTTCGTCCCCGAT | 138 |
| BCL2 | NM_000633 | TGCACCTGACGCCCTTCAC | TTCCACAAAGGCATCCCAGCC | 244 |
| CAV1 | NM_001753 | AGACTCGGAGGGACATCTCTACA | GTCATCGTTGAGGTGTTTAGGGTC | 165 |
| CPT1A | NM_001876 | CGATGTTACGACAGGTGGTTTGAC | CGGAATGTTCGGATTGATGTCGC | 200 |
| GAPDH | NM_002046 | AAGATCATCAGCAATGCCTCCTG | TGACCTTGCCCACAGCCTT | 228 |
| FSP27 | NM_022094 | ATCAGAACAGGGGACAAGGCA | CTTCACGTTCAGGCAGCCAATG | 129 |
| FOXO1 | NM_002015 | GACCTCATGGATGGAGATACATTGGA | GTGTAACCTGCTCACTAACCCTCA | 128 |
| GPX1 | NM_000581 | GAATGTGGCGTCCCTCTGAG | TGCGTTCTCCTGATGCCCA | 131 |
| HMOX | NM_002133 | GCAACAAAGTGCAAGATTCTGCC | TGGCATAAAGCCCTACAGCAACT | 140 |
| PPARA | NM_001001928 | ATTTGCTGTGGAGATCGTCCTGG | GCATCCGACTCCGTCTTCTTGAT | 214 |
| PPARG | NM_138712 | TATCCGAGGGCCAAGGCTTC | AAACCTGGGCGGTCTCCACT | 177 |
| UCP2 | NM_003355 | GATACCAAAGCACCGTCAATGCCTAC | GGCAAGGGAGGTCATCTGTCAT | 185 |
| HPRT | NM_000194 | CCTGGCGTCGTGATTAGTGATGAT | CAGAGGGCTACAATGTGATGGC | 181 |
| SDHA | NM_004168 | GCAGAACCTGATGCTGTGTG | TTCCAGAGTGACCTTCCCAGTG | 216 |
| p62 | NM_003900 | ATCTCCCGCCAGAGGCTGA | GGATGCTTTGAATACTGGATGGTGTC | 156 |
| **Primers for Rat Genes (INS1)** | | | | |
| **Gene** | **Reference** | **Forward** | **Reverse** | **Amplicon(bp)** |
| Plin2 | NM_001007144.1 | AGCCACAGATTGCGGTCG | GTCACTACATCCTTTGCCCCAG | 141 |
| Tfeb | NM_001025707.1 | GCTAACAGATGCTGAGAGCCG | CTTGAGGATGGTGCCCTTGT | 174 |
| Lamp1 | NM_012857.2 | GTTCAGCACCTCCAACTATTCCCT | CACTCTTCCACAGACCCAAACC | 174 |
| p62 | NM_175843.4 | TGAGTCGGCTTCTGCTCCAT | TCTTATCTTCTGTGCCTGTGCTGG | 184 |
| Lc3 | AY310156.1 | ACTAACCACTGCCTCTCAACCTG | TCGCTCTATAATCACCTGGGTCG | 204 |
| Ins1 | NM_019129.3 | CATCAGCAAGCAGGTCATTGTTCC | AGGTACAGAGCCTCCACCAG | 148 |
| Ins2 | NM_019130.2 | CATCAGCAAGCAGGTCATTGTTCC | GGGTGTGTAGAAGAATCCACGC | 182 |
| Pparα | NM_013196.1 | GTCATCACAGACACCCTCTCTCC | AGCCTTCACATGCGTGGAC | 165 |
| Pparγ | NM_013124.3 | GTCTCACAATGCCATCAGGTTTGG | TTTGGTCAGCGGGAAGGAC | 165 |
| Ucp2 | NM_019354.2 | CATGACAGACGACCTCCCTTG | GGTGACAAACATTACTACGTTCCAGG | 243 |

Primers were designed to efficiently anneal at 58-60*C. At least one primer in each pair spans a splice site to exclude amplification of genomic DNA. Veracity of PCR products was confirmed by appearance of single bands of indicated sizes on 1.5% agarose gel.

**Supplementary Table 3. Antibodies used in this study**

| **Primary Antibodies** | | | |
| --- | --- | --- | --- |
| **Target** | **Species** | **Dilution** | **Supplier** |
| TFEB | rabbit | 1:200 | Bethyl Laboratories, Montgomery, TX, USA |
| C-peptide | mouse | 1:100 | Meridian Life Science, Memphis, TN, USA |
| PLIN2 | guinea pig | 1:200 | Fitzgerald Industries, Acton, MA, USA |
| LAMP2 | rat | 1:100 | Abcam, Toronto, ON, Canada |
| Glucagon | mouse | 1:200 | Sigma, Oakville, ON, Canada |
| PLIN5 | guinea pig | 1:100 | ProGen, Waltham, MA, USA |
| p62 | rabbit | 1:200 | MBL, Woburn, MA, USA |
| LC3B | rabbit | 1:1000 | Cell Signaling Technologies. Danvers, MA, USA |
| Caspase-3 | rabbit | 1:500 | Santa Cruz, Dallas, TX, USA |
| Nrf2 | mouse | 1:500 | R&D, Minneapolis, MN, USA |
| β-actin | mouse | 1:10,000 | Sigma |
| **Secondary Antibodies** | | | |
| **Target** | **Species** | **Dilution** | **Supplier** |
| anti-rabbit, Alexa Fluor-488 | goat | 1:500 | Invitrogen, Waltham, MA, USA |
| anti-mouse, Alexa Fluor-488 | goat | 1:500 | Invitrogen |
| anti-rat Alexa Fluor-488 | goat | 1:500 | Invitrogen |
| anti-mouse Alexa Fluor-647 | goat | 1:500 | Invitrogen |
| anti-guinea pig, Alexa Fluor-647 | goat | 1:500 | Invitrogen |
| anti-rabbit, HRP-conjugate | goat | 1:1000 | Bio-Rad, Mississauga, ON, Canada |
| anti-mouse, HRP-conjugate | goat | 1:1000 | Bio-Rad |
